# Empirical Analysis of Health Assessment Objective and Subjective Methods on the Determinants of Health

**DOI:** 10.3389/fpubh.2022.796937

**Published:** 2022-05-09

**Authors:** Abdou Khadre Dit Jadir Fall, Florence Migot-Nabias, Najoua Zidi

**Affiliations:** ^1^Université Paris Cité, Institut de Recherche pour le Développement (IRD), Mère et Enfant en Milieu Tropical (MERIT), Paris, France; ^2^Laboratoire d'Economie Dionysien (LED), EA 3391, Université Paris VIII, Saint-Denis, France

**Keywords:** health determinant, objective and subjective health assessment methods, empirical analysis, cointegration, long-term causal relationship

## Abstract

**Background:**

There are several methods for assessing health status. The aims of this study were to investigate the empirical differences between health assessment objective and subjective methods, to identify a possible long-term relationship between methods and health determinants and the influence of these methods on the perceived level of risk according to health determinants.

**Methods:**

Using data from 1970 to 2018 in the United States, health status was assessed by perception of health, absence from work due to self-reported illness, life expectancy at birth and mortality rate. Health determinants were tobacco and alcohol consumptions, number of physicians per 1,000 persons, stay in hospitalization unit, curative care, release of greenhouse gases, per capita gross domestic product (GDP) and urbanization. The differences between health objective and subjective assessment methods were investigated through a Generalized linear model, a structural break date of health methods was investigated by Chow test and the long-term relationship between health assessment methods and health determinants by Engle and Granger cointegration test.

**Results:**

Tobacco consumption was associated with a decrease of life expectancy while no long-term causal relationship was found between them. There was a positive correlation between alcohol consumption and perception of good health with a long-term causal relationship. Although per capita GDP positively influenced life expectancy, there was no cointegration between them. The release of greenhouse gases was positively correlated with both the absence from work due to self-reported illness and the perception of good health. Finally, curative care was associated with a decrease of mortality and absence from work due to self-reported illness and an increase of life expectancy and perception of good health while hospitalization is positively correlated with mortality and negatively correlated with life expectancy with a long-term causal relationship. Finally, the number of physicians per 1,000 persons was not correlated with health assessment methods used.

**Conclusion:**

Our results highlight the influence of health assessment methods on the determinants of health and the fact that the perceived risk of health determinants changes according to the method used. Thus, the impact of health assessment methods must be considered in order to prioritize the determinants of health.

## Introduction

WHO defines health as a complete state of physical, mental and social wellbeing and not merely as the absence of disease or infirmity (1). Questions remain on how to properly measure health status. According to Ware (2), health status assessment is useful to evaluate the efficiency or effectiveness of medical interventions, the quality of care and population need. Several methods are used to assess the health status.

There are the subjective methods based on the perception of health status and self-assessment questionnaires. Generally, the perception of health is based on graduated responses such as “very good,” “good,” “average,” “bad,” and “very bad.” While the self-assessment questionnaires select one or more health dimensions such as morbidity, heart difficulties, high blood pressure… They are usually constructed using methods according to the subjects and studies. Several concepts have been developed in relation to these methods such as the subjective wellbeing. It is based on positive concepts including happiness, life satisfaction, morale, self-esteem, autonomy dimensions (3). A large number of systematic reviews have been published as regards these questionnaires measuring a specific concept in a specific population group (4–7). However, there is still a bias related to the reproducibility, reliability and validity of methods and results. Engström and Holmlund (8) showed that individuals in the low socioeconomic group tended to underestimate their need of dental care, while according to Maddox and Douglass (9), health status self-estimation is credible, effective and tends to be a better predictor of health status in the future. Wolinsky and Johnson (10) showed that seniors who report poor health are more likely to die than those who report good health. Finally, according to Barsky et al. (11), there was a correlation between the perception of health and aggregate medical morbidity, psychiatric morbidity, functional disability and hypochondriacal attitudes. These studies, despite the questions about reproducibility and reliability, showed that subjective methods can be considered as a reliable criterion in assessing health status.

Health status is, also, measured using pathological or clinical measures such as signs, symptoms, blood pressure, temperature … Indeed, health status is assessed based on reported, diagnosed diseases and the frequency of chronic diseases. Several concepts and theories have been developed in connection with this method (12). Among the concept, there is the functional ability which focused on impairment (loss or abnormality of psychological, physiological or anatomical structure or function), disability (restriction or lack of ability to perform an activity), handicap, and mental health. Mental health is measured by the SF-36 Mental Health Dimension Score indicator called MH (13–15).

There is heterogeneity in the assessment of health status. Measurement methods can be ranged from the most general evaluating biological parameters, to the most specific focused on particular aspects such as disability or mental health.

The first objective of our study was to assess the empirical differences between the two health assessment methods generally used, namely the objective and subjective methods (16, 17). The indicators for the subjective method were the perception of health status and the absence from work due to self-reported illness and the indicators for the objective method were the life expectancy at birth and the mortality rate. The influence of these methods is analyzed on the same determinants of health status selected among medical determinants (physicians per 1,000 persons, average length of stay in hospitalization units and the bed occupancy rates in curative care), non-medical determinants (alcohol and tobacco consumption), population (urbanization), economic [per capita Gross Domestic Product (GDP)], and environmental (release of greenhouse gases) variables.

The second objective of our study was to evaluate a possible existence of a long-term relationship (a correlation over time) between perception of health status, absence from work due to self-reported illness, life expectancy at birth and mortality rate, and health determinants.

For this purpose, this study used data from the United States. This choice is explained by several reasons. First of all, the United States hosts the most expensive healthcare system in the world, with strong disparities, and a health insurance system heavily dependent on employment which excludes the unemployed (18–22). Moreover, some individuals combine several jobs but are still categorized as poor, and named the “working poor” (23–25). Ross et al. (26) showed a negative relationship between older working poor and the receiving of preventive care while Miller et al. (27) showed that low income and insured men are under-diagnosed and under-treated for prostate cancer. At the opposite, Mahal et al. (28) confirmed the fact that insured men with prostate cancer were more likely to be treated and to survive compared to non-insured.

Thus, this study can target the most important determinants which emerge depending on the method used. Consequently, it would be interesting to better appreciate the individual's health status for better care and reimbursement from insurance. Moreover, the risk perceived according to the determinants of health can modify the behavior of the individual toward these determinants and consequently his general health status.

## Methods

### Study Design

The study was conducted using data, collected from databases OECD, World bank and Perspective monde, in the United States during the period 1970–2018.

Health status was evaluated by objective indicators including life expectancy at birth (average number of years that a newborn is expected to live if current mortality rates continue to apply) and mortality rate (number of deaths in the year per 1,000 people and estimated at mid-year). The subjective indicators were the absence from work due to self-reported illness (number of days lost per person per year due to an absence from work resulting for a self-reported illness) and the perception of health status (percentage of the population aged 15 and over in good health).

Non-medical health determinants were cigarette consumption [annual consumption of tobacco (cigarettes, cigars) in grams per person aged 15 and over] and alcohol consumption [annual consumption of pure alcohol (beer, wine, spirits, others) in liters per person aged 15 and over]. Health care resources were the number of physicians per 1,000 persons, the average length of stay in hospitalization units (calculated by dividing the number of bed-days by the number of discharges during the year) and the percentage of available beds in curative care (curative care). Curative care comprises health care contacts during which the principal intent is to relieve symptoms of illness or injury, to reduce the severity of an illness or injury, or to protect against exacerbation and/or complication of an illness or injury that could threaten life or normal functions.

The environmental variable was represented by the carbon dioxide emissions (metric tons per capita). Emissions were defined as the release of greenhouse gases or precursors of greenhouse gases into the atmosphere over an area and over a period of time. The calculation was made here by dividing carbon dioxide emissions in metric tons (1,000 kg) by the total number of inhabitants.

The last variables were the per capita gross domestic product (US dollars GDP/capita) and the percentage of population living in agglomerations counting more than one million residents.

### Statistical Analysis and Econometric Methods

The statistical analysis was carried out using the Gretl software version. First of all, data of health assessment methods and health determinants are presented. Then, the empirical differences between health assessment methods and health determinants were assessed using Generalized linear model (GLM) [recommended against autocorrelation and heteroscedasticity problems] which estimated the following equations:

Health status, determined by “absence from work due to self-reported illness; perception of health status; life expectancy at birth; mortality rate” is correlated to “tobacco consumption, alcohol consumption, number of physicians per 1,000 persons, average length of stay in hospitalization, bed occupancy rates in curative care, carbon dioxide emission, per capita GDP, urbanization.”

Then, the stability of health assessment methods for which data showed a change in the study period (life expectancy and mortality methods) was tested for different periods through a Chow test in order to determine a structural break date in their evolution. For this purpose, first of all, a Quandt Likelihood Ratio (QLR) test is performed in order to look for a break date. Then, a Chow test is performed using a Fisher (F) test. Finally, the means of the tested variables between before and after the break date were compared through a Mann-Whitney test.

Finally, the cointegration test was used to evaluate a long-term relationship between control variables and health assessment methods (29, 30). In this study, the Engle-Granger cointegration test between two variables was used. For this purpose, the following steps have been covered:

The stationarity (order of integration) of the variables was tested through a Dickey Full Augmented (ADF) test. The variables were differentiated in a case of no stationarity. Two variables are, potentially, cointegrated if they have the same order of integration. In this case, the Engel and Granger's method was applied to study the cointegration between the two variables. First of all, we checked that the unit root hypothesis was not rejected for the individual variables tested (step 1). Then, we checked that the unit root hypothesis was rejected for the residues of the cointegration regression (step 2 and 3) in a case of cointegration. Finally, in case of cointegration, an error correction model (ECM) was estimated with the linear variable (differentiated variable) (step 4). If the residue (e) was significant and negative, therefore, there was a long-term relationship between the two variables tested.

## Results

### Characteristics of Health Status and Determinants

According to Table 1, life expectancy at birth remained relatively moderate around 76 years in the United States despite an average annual absence from work of 5 days and a large majority of population aged 15 and over reporting being in good health (88%). A mean quantity of 2,245 g of tobacco (cigarettes, cigars) and 9 L of pure alcohol (beer, wine, spirits, others) were consumed during the study period. On average, patients spent 8 days in hospital while 2/3 of beds were available on curative care. A large part of the population (77%) lived in cities, emitted 19 tons of carbon dioxide per year with a relatively high GDP per capita (30 546 USD).

**Table 1:** Characteristics of health status and determinants of health in the United States during the 1970–2018 period.

	**Mean**	**Min–Max**
**Health assessment subjective method**		
Absence from work due to self-reported illness (Day)	4.67	3.5–5.60
Perception of health status (%)	87.88	86–88.90
**Health assessment objective method**		
Life expectancy at birth (Year)	75.77	70.90–78.90
Mortality rate (%)	8.59	7.90–9.50
**Non-medical determinants of health**		
Tobacco consumption (gram)	2245.28	1061–3606
Alcohol consumption (liter)	9.12	8.1–10.4
**Health care resources**		
Physicians per 1,000 inhabitants	2.05	1,20–2,71
Average length of stay in hospitalization (Day)	8.51	6.10–14.9
Rate of available beds in curative care (%)	67.77	61.50–78
**Economic variable**		
Per capita GDP (USD/capita)	30546.31	5234.3–65280.7
**Population**		
Urbanization (%)	77.20	73.60–82.26
**Environment variable**		
Carbon dioxide emissions (metric tons/capita)	19.35	15.50–22.51

### Association Between Health Determinants and Subjective Measurement Method

According to Table 2, the GDP per capita, the carbon dioxide emission and the stay in hospital units were positively correlated to the absence from work due to self-reported illness (*P* = 0.050*, P* = 0.026, and *P* = 0.001, respectively) while more beds available in curative care was negatively correlated with the absence from work related to self-reported illness care (*P* = 0.0001). There was a positive correlation between release of greenhouse gases and perception of health (*P* = 0.004) as well as with alcohol consumption (*P* = 0.100). For its part, tobacco did not appear to have an impact on both the absenteeism from work due to self-reported illness and the perception of health status. Finally, results showed that the perception of good health increased with the curative care (*P* = 0.008).

**Table 2:** Generalized linear model results comparing subjective and objective measurement methods of health in the United States in 1970–2018.

**Health status**	**Subjective method**		**Objective method**	
**Health determinants**	**Absence from work due to illness**	**Perception of Good health**	**Life expectancy at birth**	**Mortality rate**
Tobacco consumption	Coef (std. err.): 0.199 (0.130)	−0.010 (0.014)	−0.011 (0.006)	−0.013 (0.0511)
	*P*. value: 0.127	0.470	0.075^*^	0.791
Alcohol consumption	Coef (std. err.): – 0.116 (0.304)	0.067 (0.040)	0.0149 (0.014)	−0.096 (0.119)
	*P*. value: 0.703	0.100^*^	0.317	0.418
Physicians per 1000 persons	Coef (std. err.): 0.042 (0.173)	−0.006 (0.017)	−0.013 (0.008)	0.048 (0.068)
	*P*. value: 0.808	0.688	0.110	0.476
Stay in hospitalization units	Coef (std. err.): 0.747 (0.225)	0.045 (0.070)	−0.054 (0.011)	0.200 (0.088)
	*P*. value: 0.001^***^	0.516	0.0001^***^	0.023^**^
Available beds in curative care	Coef (std. err.): – 1.153 (0.330)	−0.106 (0.039)	0.030 (0.016)	−0.260 (0.129)
	*P*. value: 0.0001^***^	0.008^***^	0.060^*^	0.045^**^
Per capita GDP	Coef (std. err.): 0.187 (0.095)	0.006 (0.021)	0.023 (0.004)	−0.040 (0.037)
	*P*. value: 0.050^**^	0.762	0.0001^***^	0.273
Urbanization	Coef (std. err.): – 1.167 (1.289)	0.358 (0.630)	0.013 (0.063)	0.237 (0.504)
	*P*. value: 0.365	0.570	0.831	0.638
Carbon dioxide emission	Coef (std. err.): 0.568 (0.255)	0.121 (0.042)	−0.018 (0.012)	0.004 (0.043)
	*P*. value: 0.026^**^	0.004^***^	0.129	0.912

*** *P ≤ 0.01*,

** *P ≤ 0.05*,

* *P ≤ 0.10; In bold, significant P value*.

### Association Between Health Determinants and Objective Measurement Method

The stay in hospitalization units was negatively correlated with life expectancy at birth as well as tobacco consumption (*P* = 0.0001 *and P* = 0.075, respectively) while there was a positive correlation between the availability of bed in curative care as well as per capita GDP and life expectancy (*P* = 0.060 *and P* = 0.0001, respectively). More beds available in curative care was negatively correlated with the mortality rate while there was a positive correlation between the stay in hospitalization units and the mortality rate (*P* = 0.045 *and P* = 0.023, respectively).

### Structural Break Tests

As shown in Table 3, QLR test showed a break date in 2001 regarding the life expectancy at birth, confirmed by the Chow test. The Mann—Whitney *U* test confirmed an increase of the life expectancy after 2001 through the mean comparison between the two periods. Indeed, before the break date, the mean age of life expectancy was 74 and 78 years after. The same pattern was observed with mortality rate through a decrease of the mortality rate after the break date 2000.

**Table 3:** Chow test in the period 1970–2018.

	**Life expectancy at birth**	**Mortality rate**
Breaking point identified by the QLR test	2001 *F*_(9,21)_ = 6.088	2000 *F*_(9,21)_ = 4.897
Chow test	*F*_(9,21)_ = 6.088	*F*_(9,21)_ = 4.897
	P *F*_(9,21)_ > 6.0851 = 0.0003^^***^^	P *F*_(9,21)_ > 4.897 = 0.0012^^***^^
Mean comparison	Mean 1970–2000: 74.37	Mean 1970–1999: 8.79
	Mean 2002–2018: 78.25	Mean 2001–2018: 8.26
Mann–Whitney *U* test	*P*<0.0001^^***^^	*P*<0.0001^^***^^

*** *P ≤ 0.01; In bold, significant P value*.

### Long-Term Relationship Between Health Assessment Methods and Health Determinants

In Tables 4–6, only variables with the same order of integration, so a possibility of a long-term relationships, were shown.

**Table 4:** Engle Granger cointegration test between health status perception and alcohol consumption and bed occupancy rate in the period 1982–2018.

	**Ln perception of health status**	**Ln alcohol consumption**	**Ln available beds in curative care**
**Dickey-Fuller tests on first difference**			
**Null hypothesis of unit root: a = 1**			
**Test without constant**			
T statistics	−4.920	−3.078	−3.897
*P*	*P* < 0.0001	0.002	*P* < 0.0001
**Test with constant**			
T statistics	−4.877	−3.070	−3.970
*P*	0.0003	0.028	0.0015
**Test with constant and time trend**			
T statistics	−4.947	−3.076	−3.975
*P* value	**0.0016** ^^***^^	0.111	**0.009** ^^***^^
**Engel and Granger cointegration test**			
**Step 1: Testing a unit root**			
Without constant	*P* value: 0.908	*P* value: 0.528	*P* value = 0.336
**Step 2: cointegration regression**			
Dependent variable		Coef. 2.054	Coef. 1.071
Health status perception		***P*** value: <0.0001^^***^^	***P*** value: <0.0001^^***^^
**Step 3: Dickey-Fuller regression**			
Lag 1			
Null hypothesis of unit root: a = 1		**P. asymptotic value: 0.045** ^^**^^	**P. asymptotic value: <0.0001** ^^***^^
**Step 4: Error correction model**			
Dependent variable		First difference ln alcohol consumption:	First difference ln Available beds in curative care
First difference Health status perception		*Coefficient:* −0.011	*Coefficient:* −0.00013
		*P* value: 0.707	*P* value: 0.804
		**e_1**	**e_1**
		*Coefficient:* **−0.347**	*Coefficient:* **−0.345**
		*P* value: 0.004^^***^^	*P* value: 0.007^^***^^
		d_lnpes_1	d_lnpes_1
		*Coefficient:* 0.345	*Coefficient:* 0.199
		*P* value: 0.026^^**^^	*P* value: 0.180

*** *P ≤ 0.01*,

** *P ≤ 0.05; In bold, significant P value*.

Absence from work due to illness, stay in hospitalization units, curative care and per capita GDP series were all integrated in order 1. However, according to the Engle and Granger cointegration test, there was not a long-term relationship between absence from work due to illness and the health determinants stay in hospitalization units, curative care and per capita GDP series (results not shown).

Even, according to Table 4, there was a long-term relationship between perception of health status and curative care (*P* value of Dickey-Fuller regression <0.0001) as well as alcohol consumption (*P* value of Dickey-Fuller regression = 0,045). Correction Error Model showed a negative and significant coefficient (e) for both.

Engle and Granger cointegration test showed a cointegration between mortality rate and curative care (*P* value of Dickey Fuller regression = 0.038) while the Correction Error Model showed a negative but not significant coefficient (e). Thus, there was not a long-term causal relationship between the mortality rate and curative care (Table 5).

**Table 5:** Engle Granger cointegration test between mortality rate and bed occupancy rate in the period 1970–2018.

	**Ln mortality rate**	**Ln available beds in curative care**
**Augmented Dickey-Fuller tests on first difference** **Null hypothesis of unit root: a = 1**		
**Test without constant**		
T statistics	−8.259	−3.897
*P* value	***P*** < 0.0001^^***^^	***P*** < 0.0001^^***^^
**Test with constant**		
T statistics	−8.278	−3.970
*P* value	***P*** < 0.0001^^***^^	**0.0015** ^^**^^
**Test with constant and time trend**		
T statistics	−8.652	−3.975
*P* value	***P*** < 0.0001^^***^^	**0.009** ^^***^^
**Engel and Granger cointegration test**		
**Step 1: Testing a unit root**		
Without constant	*P* value: 0.314	*P* value: 0.336
**Step 2: Cointegration regression**		
Dependent variable		Coef. 0.510
Mortality rate		***P*** value: <0.00001^^***^^
**Step 3: Dickey-Fuller regression**		
Lag 1 Null hypothesis of unit root: a = 1		**P. asymptotic value: 0.038** ^^***^^
**Step 4: Error correction model**		
Dependent variable		First difference ln available beds in curative care:
First difference LN mortality rate		*Coefficient:* −0.001
		*P* value: 0.320
		**e_1**
		*Coefficient:* −0.112
		*P* value: 0.109
		d_lnmortality rate_1
		*Coefficient:* −0.137
		*P* value: 0.340

*** *P ≤ 0.01*,

** *P ≤ 0.05; In bold, significant P value*.

Finally, cointegration test showed a long-term causal relationship between the life expectancy and stay at hospitalization unit. Indeed, the *P* value of Dickey Fuller regression was significant (*P* = 0.002) and the residue (e) was negative and significant (Table 6).

**Table 6:** Engle Granger cointegration test between life expectancy and stay in hospitalization unit in the period 1970–2018.

	**Ln life expectancy**	**Ln stay in hospitalization unit**
**Augmented Dickey-Fuller Tests on First difference** **Null hypothesis of unit root: a = 1**		
**Test without constant**		
T statistics	−2.447	−3.089
	0.013	0.002
**Test with constant**		
T statistics	−5.935	−3.520
*P value*	***P*** < 0.0001^^***^^	**0.011** ^^***^^
**Test with constant and time trend**		
T statistics	−6.815	−3.704
*P value*	***P*** < 0.0001^^***^^	**0.031** ^^***^^
**Engel and Granger cointegration test**		
**Step 1: Testing a unit root**		
With constant	***P*** value: 0.020^^**^^	*P* value: 0.430
**Step 2: Cointegration**		
Dependent variable	Const (*p* value) of regression: 4.575	Coef. −0.118
Life expectancy at birth	**(<0.00001** ^^***^^ **)**	***P*** value: <0.00001^^***^^
**Step 3: Dickey-Fuller regression**		
Lag 1 Null hypothesis of unit root: a = 1		**P. asymptotic value: 0.095** ^*^
**Step 4: Error correction model**		
Dependent variable		First difference ln stay in hospitalization:
First difference life expectancy		*Coefficient:* −0.061
		*P* value: 0.002^^***^^
		e_1
		*Coefficient:* –**0.148**
		*P* value: **0.076**
		d_life expectancy_1
		*Coefficient:* 0.146
		*P* value: 0.309

*** *P ≤ 0.01*,

** *P ≤ 0.05*,

* *P ≤ 0.10; In bold, significant P value*.

## Discussion

This article investigated the empirical differences between health assessment objective and subjective methods on health determinants and a possible existence of a long-term relationship between them in the United States.

We found an increase of the life expectancy and a decrease of the mortality rate in the 2000s with specific dates highlighted by QLR, Chow and Mann-Whitney tests. These results were in line with those of Woolf and Schoomaker (31) and Mokdad et al. (32). However, this increase in life expectancy is not as elevated as in countries such as France or the United Kingdom. Access to healthcare may play a key factor insofar as in the United States, this access is conditioned by employment. Despite the fact that European countries have a high per capita GDP, we can hypothesize an influence of GDP in these countries but not in the United States.

Indeed, Swift (33) estimated that total GDP and per capita GDP exerted a significant influence on life expectancy for most European countries (1% increase in life expectancy resulting from 6% increase in total GDP) while according to Zaman et al. (34), the relationship between GDP and life expectancy may be explained by a direct relationship between GDP and health government expenditure. However, in the United States, government's intervention is limited. Thus, the finding that there was no long-term relationship between life expectancy and per capita GDP (results not shown) in the United States was in line with this. Moreover, the United States is the richest state in the world but it remains far behind other states in terms of life expectancy, ranging for example at the 18th position for life expectancy of women, among the 30 OECD (Organization for Economic Co-operation and Development) countries, slightly above Greece, Korea, and Mexico (35).

Our results showed that life expectancy in the United States was negatively associated with tobacco consumption while there was not a long-term causal relationship between them in the period 1970–2018 (results not shown). Indeed, many papers concluded that the most relevant health indicators for poor populations from the United states were obesity, alcohol and tobacco consumption (35, 36). Holford et al. (37) estimated 17.6 million deaths related to smoking from 1964 to 2012, in the continuity of what Rogers and Powell-Griner (38) described formerly with a higher life expectancy for no smokers compared to former smokers and for former smokers compared to current smokers. Mokdad et al. (32) considered tobacco consumption, high body mass index and alcohol consumption as the top risk factors for diminished life expectancy.

Compared to European countries, tobacco consumption is a concern, although it remains moderate in Scandinavian countries (17% in Denmark, 16% in Finland, 15% in Norway, and 11% in Sweden) to which is attributed a reputation for better health systems in link with better levels of health indicators (39).

The results showing a long-term positive relationship between alcohol consumption and perception of health may be related to the indicator used and also the personal beliefs. Thus, according to Chang et al. (40), 80% of people in Helsinki believe that drinking red wine is healthier than drinking beer or spirits. Also, Strandberg et al. (41) reported that red wine drinkers had a 34% lower mortality rate than beer or vodka drinkers. So, on the one hand, there is a popular belief in the health benefits of certain types of alcohol and on the other, the fact that no alcohol consumption can prevent serious illnesses related to its consumption, therefore for avoiding early mortalities. This paradox was a perfect illustration of the differences found for some health determinants according to the method used for their assessment. The same pattern was found for the influence of releasing greenhouse gases on the absence from work due to self-reported illness and on the perception of health status.

On the one hand, the finding of a positive effect may be explained by the fact that individuals move, go to their occupation (work, leisure) therefore improve their wellbeing because they feel healthy. On the other hand, a negative effect may be deduced in case of illness due to pollution, that will prevent people from working and hence increase their absence from work due to self-reported illness.

The finding (i) that the stays at hospitalization unit was negatively associated with life expectancy and positively associated with mortality rate and (ii) that curative care was related to increased life expectancy and perception of good health and to decreased mortality and absence from work due to self-reported illness as well as was rather expected. Over the long term, receiving curative care can reduce the severity of an illness or injury, protect against exacerbation and/or complication of an illness or injury while the longer a hospitalization stay is, the greater the risk of mortality, as reflecting a serious case of concern. Moreover, the finding that receiving curative care influenced perception of health care status was explained by the positive virtues of receiving curative care.

### Limitation

It may be interesting to conduct this study in a multi-country panel to confirm the results observed in the United States or to constitute a comparative panel between poor and rich countries in order to compare the influence of health determinants according to the status of countries. Also, cointegration studies are more interesting over long periods.

## Conclusion

To our knowledge, no work previously investigated the empirical differences between health assessment objective and subjective methods with a long-term causal relationship analysis. Our results highlight the fact that the determinants of health change according to the health status assessment method used, with an impact on the long-term relationship between health indicators of the methods and health determinants. Non-medical determinants were the most affected. For examples, tobacco consumption was associated to a decrease of life expectancy but there was not a long-term relationship while there was a positive correlation between alcohol consumption and perception of good health with a long-term causal relationship. Air pollution was positively correlated to absence from work du to self-reported illness and perception of good health while there were no long-term relationships between them. In contrast, whatever the methods, medical determinants play an important role. Thus, the impact of health assessment methods must be considered in order to prioritize the determinants of health.

## Data Availability Statement

The original contributions presented in the study are included in the article/supplementary material, further inquiries can be directed to the corresponding author/s.

## Author Contributions

AF, FM-N, and NZ wrote the article. All authors reviewed the manuscript and approved it for publication.

## Conflict of Interest

The authors declare that the research was conducted in the absence of any commercial or financial relationships that could be construed as a potential conflict of interest.

## Publisher's Note

All claims expressed in this article are solely those of the authors and do not necessarily represent those of their affiliated organizations, or those of the publisher, the editors and the reviewers. Any product that may be evaluated in this article, or claim that may be made by its manufacturer, is not guaranteed or endorsed by the publisher.
